# Improved Position Estimation Algorithm of Agricultural Mobile Robots Based on Multisensor Fusion and Autoencoder Neural Network

**DOI:** 10.3390/s22041522

**Published:** 2022-02-16

**Authors:** Peng Gao, Hyeonseung Lee, Chan-Woo Jeon, Changho Yun, Hak-Jin Kim, Weixing Wang, Gaotian Liang, Yufeng Chen, Zhao Zhang, Xiongzhe Han

**Affiliations:** 1 College of Electronic Engineering, South China Agricultural University, Guangzhou 510642, China; gaopeng.peng@stu.scau.edu.cn (P.G.); weixing@scau.edu.cn (W.W.); 201621190216@stu.scau.edu.cn (G.L.); chenyufeng@stu.scau.edu.cn (Y.C.); 2 Department of Biosystems Engineering, College of Agriculture and Life Sciences, Kangwon National University, Chuncheon 24341, Korea; hslee91@kangwon.ac.kr; 3 Interdisciplinary Program in Smart Agriculture, College of Agriculture and Life Sciences, Kangwon National University, Chuncheon 24341, Korea; 4 Department of Biosystems Engineering, College of Agriculture and Life Sciences, Seoul National University, Seoul 08826, Korea; wwemania91@snu.ac.kr (C.-W.J.); changho288@snu.ac.kr (C.Y.); 5 Global Smart Farm Convergence Major, College of Agriculture and Life Sciences, Seoul National University, Seoul 08826, Korea; 6 Research Institute of Agriculture and Life Science, Seoul National University, Seoul 08826, Korea; 7 Key Laboratory of Smart Agriculture System Integration, Ministry of Education, China Agricultural University, Beijing 100083, China; zhaozhangcau@cau.edu.cn; 8 Key Laboratory of Agriculture Information Acquisition Technology, Ministry of Agriculture and Rural Affairs of China, China Agricultural University, Beijing 100083, China

**Keywords:** Kalman filter (KF), inertial measurement unit (IMU), global navigation satellite system (GNSS), autoencoder neural network, agricultural mobile robots (AMRs)

## Abstract

High-precision position estimations of agricultural mobile robots (AMRs) are crucial for implementing control instructions. Although the global navigation satellite system (GNSS) and real-time kinematic GNSS (RTK-GNSS) provide high-precision positioning, the AMR accuracy decreases when the signals interfere with buildings or trees. An improved position estimation algorithm based on multisensor fusion and autoencoder neural network is proposed. The multisensor, RTK-GNSS, inertial-measurement-unit, and dual-rotary-encoder data are fused with Extended Kalman filter (EKF). To optimize the EKF noise matrix, the autoencoder and radial basis function (ARBF) neural network was used for modeling the state equation noise and EKF measurement equation. A multisensor AMR test platform was constructed for static experiments to estimate the circular error probability and twice-the-distance root-mean-squared criteria. Dynamic experiments were conducted on road, grass, and field environments. To validate the robustness of the proposed algorithm, abnormal working conditions of the sensors were tested on the road. The results showed that the positioning estimation accuracy was improved compared to the RTK-GNSS in all three environments. When the RTK-GNSS signal experienced interference or rotary encoders failed, the system could still improve the position estimation accuracy. The proposed system and optimization algorithm are thus significant for improving AMR position prediction performance.

## 1. Introduction

With the development of precision and smart agriculture, agricultural mobile robots (AMRs) have been widely applied in many fields [1], such as planting, weeding, picking, and field transportation. The requirements of AMRs have increased with labor costs, and the variety of AMRs used have different application conditions. Jeon et al. [2] developed a fully autonomous tillage tractor that could engage in path planning under given field conditions. Mahmud et al. [3] designed a multi-objective AMR path planning algorithm with an optimized routing strategy using the genetic algorithm; this system reduced both the running time and service cost of the AMR. Aravind et al. [4] noted that although AMRs are effective equipment for improving productivity without sacrificing the quality of agricultural products, the application effects differ based on the environment.

AMRs can be used for special functions by integrating various sensors to collect multidimensional information to construct sensor-motion feedback systems [5]. Zhang et al. [6] proposed a quadratic traversal algorithm to solve the weeding path planning problem using an AMR in a cornfield; the convolutional neural network (CNN) was applied to this system to quickly recognize weeds and generate the target edges. The weed contours were reflected in three-dimensional coordinates, and the targets were converted to motion control instructions for the AMR. Although the recognition accuracy reached 90%, this study did not consider the motion attitudes and directions of the AMR in dynamic and open external environments. With its limited anti-interference capability, an AMR would have trouble filtering environmental noise in real-time and may deviate from the expected path. In extreme cases, the degree of deviation of the AMR may increase gradually; thus, the AMRs may deviate completely from their expected trajectories. Therefore, it is necessary for the AMRs to correctly execute control instructions based on multisensor data collected from real-time motion states.

Nowadays, global navigation satellite systems (GNSSs), such as global positioning system (GPS), Beidou, Galileo, and Glonass, are widely used in military, agricultural, vehicle navigation, and other applications [7]. The positioning accuracy of the traditional GPS is about 10 m, while this accuracy could reach 2 m with the help of a mobile communication station [8]. With the development of various global satellite positioning systems, the positioning accuracy of the currently available GNSS is about 20–100 cm without assistance from other methods [9]. However, GNSS is still not enough to cover the requirement in high accuracy and automatic positioning scenarios, such as agricultural seeder and rice transplanter requiring more accurate positioning. As a common assistant positioning method, real-time kinematic GNSS (RTK-GNSS) is a dynamic differential positioning scheme based on high-accuracy carrier-dependent observations [10]. Thus, the positioning accuracy of the RTK-GNSS is about 1–3 cm. Emmi et al. [11] studied a fused AMR based on an RGB camera, an inertial measurement unit (IMU), and the GNSS to achieve fast recognition of specific targets. However, both the GNSS and RTK-GNSS rely on open-air measurements to achieve high-accuracy positioning. Kanagasingham et al. [12] developed a paddy field weeding robot with the help of a compass and GPS; the equipment was tested in a complex environment with a positioning accuracy of 100 mm. With the help of a vision system, the weeding robot could follow a predetermined path without damaging the plants. This study also showed that the resolution accuracy of the vision system may decrease with an increase in weed concentrations, thereby causing path deviations of the weeding robot. Considering that AMRs are often operated in complex environments covered by plants when the GPS signals are poor, the AMRs may deviate from their target paths, which negatively influences the tasks, in addition to the target detection camera and weeding machinery. Therefore, it is necessary to develop an AMR with accurate position prediction ability even when the satellite signals are affected. 

The key to achieving high-accuracy position predictions for AMRs is multisensor data fusion. GPSs cannot provide high-accuracy positioning data when the satellite signals experience interference from buildings or trees. However, IMU and other motion sensors can work independently without being influenced by the external environment, whereas their accumulated errors may increase over time. Therefore, it is important to facilitate complementary data fusion between the GNSS and attitude sensors. Data fusion is widely used in multisensor situations [13] to achieve high-accuracy position estimations. Yazdkhasti et al. [14] proved that the Kalman filter (KF) is an effective multisource sensor data fusion algorithm for navigation sensors; thus, KF can be applied to multisensor fusion.

Zhang et al. [15] proposed a pipeline mapping system based on the KF to fuse data from IMU and odometer sensors. In this study, the KF was used for multisensor data fusion to improve the horizontal and vertical positioning accuracies by 43% and 57%, respectively, for pipeline mapping and positioning test progress. The results showed that the KF played an important role in improving sensor measurement performance. Han et al. [16] developed a low-cost GPS/IMU fusion system for position estimation. The IMU sensor multigroup GPSs were used to detect motion attitude data to build the multisensor fusion system based on KF. The position prediction accuracy of the system was 0.778 m for straight and 0.429 m for curved paths, which were 70.6% and 72.5% higher than those obtained with a single GPS, respectively. However, the GPS in this study supported only a single-band satellite signal with limited positioning accuracy. At the same time, the noise variance matrix of the KF algorithm was optimized using statistical analysis with fixed values in the experiments, whereas the noise of the KF may change dynamically in different environments. According to Ryu et al.’s [17] research, the KF may not be able to effectively follow time-varying or unknown parameters owing to the influence of environmental noise, especially in nonlinear systems. Thus, noise optimization of the KF is important to improve its position estimation performance. The KF noise exists mainly in the state and measurement equations, e.g., state noise during uniform motion and measurement noise of the GPS and other sensors [18], and is time-varying and nonlinear. 

The commonly used methods to determine the KF noise are empirical estimations and statistical analysis. Yousuf et al. [19] designed an IMU/GPS/odometer fusion system based on the KF. The noise value of the state equation was set as 0.1, and the noise of the measurement equation was set as the standard deviation of the sensors. Hu et al. [20] proposed an adaptive KF algorithm based on covariance estimation of the state noise. The algorithm was based on the maximum likelihood (ML) of realizing dynamic updates, and the state noise was estimated with a large number of samples. Moreover, a fixed-length memory window was introduced to the algorithm to reduce its relative iteration time by 15.03%, while the position estimation accuracy improved by 33.5%. This research proved that it is crucial to optimize KF noise to improve position estimation accuracy.

In recent years, neural networks have been applied in many fields [21]. With their nonlinear activation function, multilayer neural network, and back-propagation characteristics, machine-learning and deep-learning methods have important influences on feature extraction and nonlinear fitting. Pesce et al. [22] constructed the radial basis function (RBF) to improve the anti-interference performance of the KF. The simulation results showed that the optimized KF had better filtering robustness, although the study lacked performance testing under complex environmental conditions. Zhao et al. [23] proposed an adaptive KF indoor positioning algorithm based on CNN. The CNN extracted the state features and measurement noise of the KF, which was then applied to position estimation. With the optimized algorithm, the positioning accuracy improved by 22%, showing that the CNN approach had good effects on the noise parameter optimization of the KF algorithm. However, the study adopted only single-source data without multisensor fusion, because of which the positioning accuracies may reduce in complex environments.

Hence, the KF algorithm proposed in this work is based on multisensor fusion and neural networks for the position prediction of AMRs. The KF was used to fuse multisensor data, and the neural network was designed to optimize the noise parameter of the KF to improve position estimation accuracy. The main contributions of this work are as follows:

(1) An AMR test platform was built and integrated with an IMU/RTK-GNSS/rotary encoder to measure multidimensional motion data.
(2) The multisensor fusion algorithm was constructed using the KF method.
(3) A neural network was proposed based on the multisensor fusion algorithm to optimize the noise parameters of the KF equations.
(4) The performance of the proposed algorithm was evaluated via various experiments.

## 2. Materials and Methods

### 2.1. AMR Description

**Figure 1:**
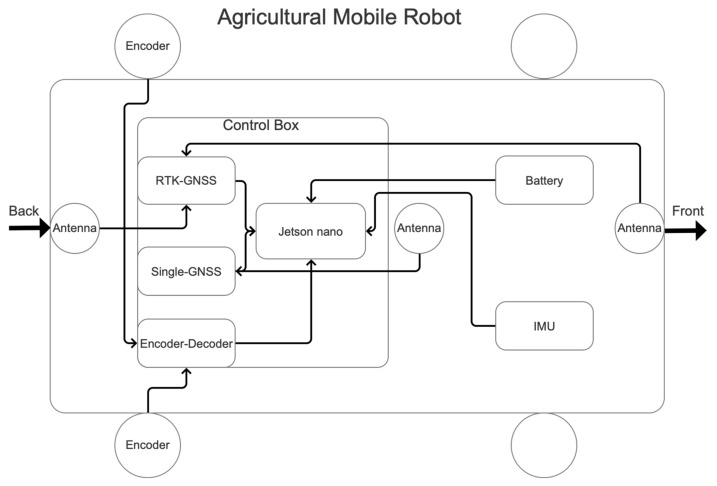
Architecture of the AMR consisting of multiple sensors and a data processing terminal.

**Figure 2:**
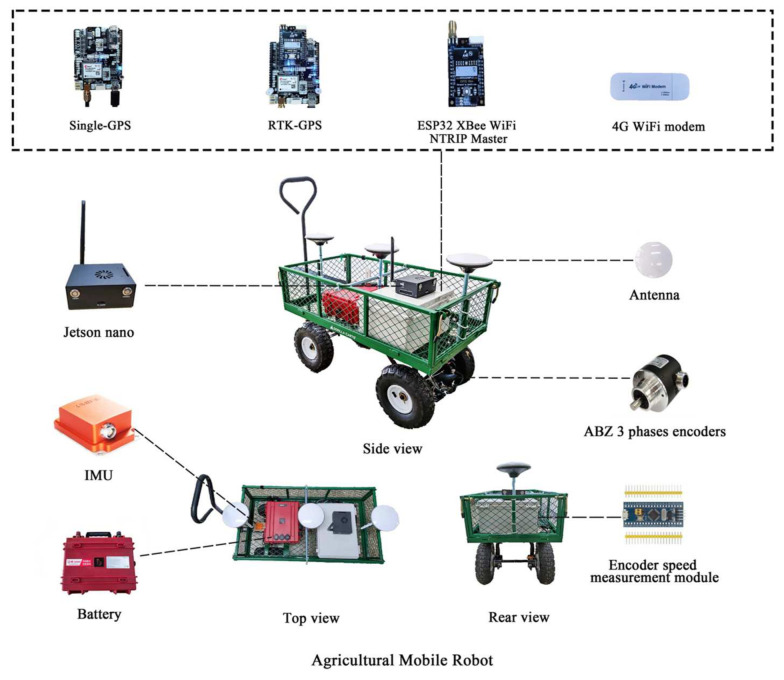
Assembled sensors and components of the AMR.

The framework of the AMR used in this study is shown in Figure 1. The GOR400 (Gorilla Carts, Eden Prairie, MN, USA) was used as the AMR test platform. Its tire diameter is 25.4 cm, and the total length, width, and height are 86.4 cm, 45.7 cm, and 94 cm, respectively. The weight of the AMR is 17 kg. The assembled AMR with its sensors is shown in Figure 2.

To accurately measure the motion attitude of the AMR, the MTI-30 (Xsens Co., Ltd., Enschede, The Netherlands) IMU was used in this work. The IMU contains a 3-axis accelerometer, 3-axis gyroscope, and 3-axis magnetometer with attitude fusion functionality. The output accuracies of the roll, pitch, and yaw are 0.2°, 0.5°, and 1°, respectively. The dual GNSS was constructed with simpleRTK2B (Ardusimple Co., Ltd., Lleida, Spain) and simpleRTK2B lite (Ardusimple Co., Ltd., Lleida, Spain). Based on the dual GNSS, the ESP32 XBee module, which contains network transport of RTCM via internet protocol (NTRIP) (Ardusimple Co., Ltd., Lleida, Spain) were integrated as the RTK-GNSS to provide high-accuracy positioning data. A 4G WiFi modem (A701, IEASUN Co., Ltd., Shenzhen, China) was used to provide access to the Internet to get corrected positioning data for RTK-GNSS. Further, the simpleRTK2B with a single antenna was used as a single-GNSS for comparison with the RTK-GNSS. The simpleRTK2B is based on the positioning chip (ZED-F9P, Ublox, Thalwil, Switzerland), which has the advantages of fast positioning and strong anti-interference ability. To improve the positioning performance of the simpleRTK2B, a calibrated survey GNSS antenna (Ardusimple Co., Ltd., Lleida, Spain) was used in the AMR, which supported GNSS as well as the Beidou, Glonass, and Galileo positioning systems. To collect real-time motion speeds of the AMR, dual rotary encoders E50S8-3000 (Autonics Co., Ltd., Seoul, Korea) were installed on the two rear wheels of the AMR. The encoders were established with rigid connections using ARM through the gears, which could output 3000 pulses per revolution (PPR); the maximum allowable PPR is 5000 revolutions per minute (rpm). Thus, the encoders not only meet the speed measurement requirements of the AMR but also provide good measurement accuracies. The above sensors were connected to the processing core, i.e., the Jetson Nano (NVidia Co., Ltd., Santa Clara, CA, USA) development kit. The Jetson Nano was used as the data collection and processing terminal and was connected to each sensor through USB serial ports to transmit data. The power supplies of all the AMR components were supported by the IP33120A-RED (E-power Co., Ltd., Ansan-si, Korea) battery.

The RTK-GNSS and single-GNSS data output baud rate was 9600 bps, transmitting data to the Jetson Nano via a USB-to-TTL line. The positioning data frame from RTK-GNSS and single-GNSS was $GNRMC with time, calibration result, longitude, latitude, ground speed, etc., of which the data output frequency was 10 Hz. The output frequency and baud rate of the IMU were 50 Hz, and 115200 bps with data frames $PRDID and $PSONCMS. The $PRDID provided the roll, pitch, and yaw data of the AMR, while the $PSONCMS provided the 3-axis acceleration, 3-axis angular velocity, and 3-axis magnetometer details. The applied rotary encoders belonged to passive output equipment; thus, the encoder speed measurement module, i.e., STM32F103C8T6 development board (STMicroelectronics Group, Shenzhen, China) was used to handle the output pulse. The STM32F103C8T6 was set in the encoder working mode to count and convert the total pulses. The data output frequency of the STM32F103C8T6 was 2 Hz, and its baud rate was 115,200 bps. To reduce errors due to speed, the mean of the speed values of the two encoders was calculated as the speed of the AMR. The single-wheel speed is calculated as follows:

(1) $$V=2\pi \cdot r\cdot \frac{\;\mathrm{TotalPulse}\;}{\;\mathrm{PPR}\cdot T\;}$$

where V is the wheel speed, m⋅s^−1^; r is the wheel radius, m; T is the period of data output which is 0.5 s; and TotalPulse denotes the captured total pulse of the rotary encoder.

### 2.2. Establishment of the Multisensor Data Fusion Model 

As a recursive optimal estimation algorithm, the KF mainly includes the prediction and update processes [24], as shown in Equations (2) and (3). The ${\hat{x}}_{t}^{-}$ and ${\hat{x}}_{t-1}$ denote prior and posterior estimations, respectively; $H_{t}$ and $F_{t}$ denote measurement sensitivity matrix and state transition matrix, respectively; $P_{t}^{-}$ and $K_{t}$ represent the covariance and gain of the KF, which includes the state noise matrix $Q_{t}$ and measurement noise matrix $R_{t}$. The optimization of **Q** and **R** has a significant impact on the KF performance. The matrix determination method [25] was used to calculate all the standard deviations (Std) of each dimension of the noise matrices independently. **Q** and **R** represent the degrees of confidence between the predicted value and measured data, i.e., **Q** and **R** may change the weights of the predicted and measured values by impacting $K_{t}$. Therefore, reasonable **Q** and **R** matrices are important for improving the prediction performance of the KF.

(2) $$\mathrm{Predict}:\left\{\begin{array}{c} {\hat{x}}_{t}^{-}=F_{t}{\hat{x}}_{t-1}\\ P_{t}^{-}=F_{t}P_{t-1}F_{t}^{T}+Q_{t} \end{array}\right.$$

(3) $$\mathrm{Update}\;:\left\{\begin{array}{c} K_{t}=P_{t}^{-}H_{t}^{T}{\left(H_{t}P_{t}^{-}H_{t}^{T}+R_{t}\right)}^{-1}\\ {\hat{x}}_{t}={\hat{x}}_{t}^{-}+K_{t}\left(z_{t}-H_{t}{\hat{x}}_{t}^{-}\right)\\ P_{t}=\left(I-K_{t}H_{t}\right)P_{t}^{-}{\left(I-K_{t}H_{t}\right)}^{T}+K_{t}R_{t}K_{t}^{T} \end{array}\right.$$

**Figure 3:**
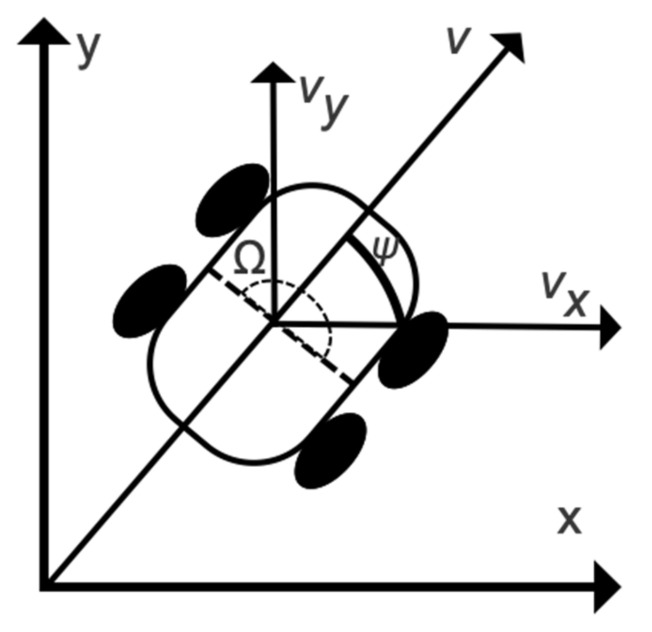
Motion model of the AMR.

Considering that the RTK-GNSS is influenced by buildings and trees, the RTK-GNSS, IMU, and rotary encoders should be fused [26] to establish the state and measurement equations of the KF. The multisensor fusion algorithm combines position, attitude, and speed information. The AMR was thus simplified as a two-dimensional motion model [27], as shown in Figure 3.

According to the AMR motion model, the vector of the state equation is defined as $X_{k}={\left[px_{k},py_{k},\psi_{k},v_{k},\Omega_{k},a_{k}\right]}^{⊤}$, where px,py represents the universal transverse Mercator (UTM) position coordinates of the AMR; and ψ, v, Ω, and a denote the heading angle, speed, yaw-rate, and acceleration of the AMR, respectively, and the Ω is calculated by differential operation of ψ to obtain AMR’s motion state and trend. As the motion equation of AMR is nonlinear, the sensor data were fused with an extended Kalman Filter (EKF) according to Goncalves et al.’s research method [28]. Each dimensional element of the state vector can be described with nonlinear formulas [29] as follows:

(4) $$\left\{\begin{array}{c} px_{k+1}=px_{k}+v_{k}\cos\left(\psi_{k}\right)\Delta t\\ py_{k+1}=py_{k}+v_{k}\sin\left(\psi_{k}\right)\Delta t\\ v_{k+1}=a_{k}\Delta t+v_{k}\\ \psi_{k+1}=\psi_{k}+\Omega_{k}\Delta t\\ \Omega_{k+1}=\Omega_{k}+\omega_{\Omega k}\\ a_{k+1}=a_{k}+\omega_{ak} \end{array}\right.$$

where $\omega_{\Omega k}$ and $\omega_{ak}$ denote the noise components of the yaw-rate and acceleration, respectively, and Δt is the time step of state updating. Thus, the matrix formula of the AMR is obtained as

(5) $$x_{k+1}=\left[\begin{array}{cccccc} 1 & 0 & 0 & \Delta t\cos\left(\psi_{k}\right) & 0 & 0\\ 0 & 1 & 0 & \Delta t\sin\left(\psi_{k}\right) & 0 & 0\\ 0 & 0 & 1 & 0 & \Omega \Delta t & 0\\ 0 & 0 & 0 & 1 & 0 & \Delta t\\ 0 & 0 & 0 & 0 & 1 & 0\\ 0 & 0 & 0 & 0 & 0 & 1 \end{array}\right]x_{k}+\omega_{xk}$$

where $\omega_{xk}$ is the noise of state equation, assuming that each state vector is statistically independent and the state equation noise follows the Gaussian distribution on (0, **Q**). For the established AMR model, the measurement equation is as follows:

(6) $$z_{k}=\left[\begin{array}{cccccc} 1 & 0 & 0 & 0 & 0 & 0\\ 0 & 1 & 0 & 0 & 0 & 0\\ 0 & 0 & 1 & 0 & 0 & 0\\ 0 & 0 & 0 & 1 & 0 & 0\\ 0 & 0 & 0 & 0 & 0 & 1 \end{array}\right]x_{k}+\omega_{zk}$$

where $z_{k}={\left[x,y,\psi ,v,a\right]}^{⊤}$; x,y represent the measured position coordinates; ψ is the yaw angle obtained from the IMU sensor, $\omega_{zk}$ is the noise of the measurement equation, which is derived mainly from the drift noise of the sensors and stochastic noise of the external environment [30] following the Gaussian distribution on (0, **R**).

### 2.3. Optimization of Noise Matrix for ***Q*** and ***R***

Research has shown that the noise matrices **Q** and **R** of the state and measurement equations have important effects on the performance of the EKF. With the development of deep-learning methods, applications in the field of data optimization are gradually increasing [31]. As an important branch of deep learning, the unsupervised learning model has great significance for feature extraction and modeling of unlabeled data. Labeling and structuring data are complex tasks, which provide important bases for unsupervised learning to process unlabeled data. Ning et al. [32] developed a neural network based on the RBF to improve the navigation accuracy of the EKF in GNSS and IMU integration systems in complex urban environments; with the help of the RBF neural network, the developed algorithm improved the position prediction accuracy of EKF when the GNSS signal was interrupted. Park et al. [33] established a KF noise estimation algorithm based on denoising the autoencoder network; the core of this method involved obtaining a newly constructed sequence after feature extraction through the autoencoder. The autoencoder was designed to extract features of and denoise the KF. This study improved the accuracy of KF for effective battery voltage estimation.

**Figure 4:**
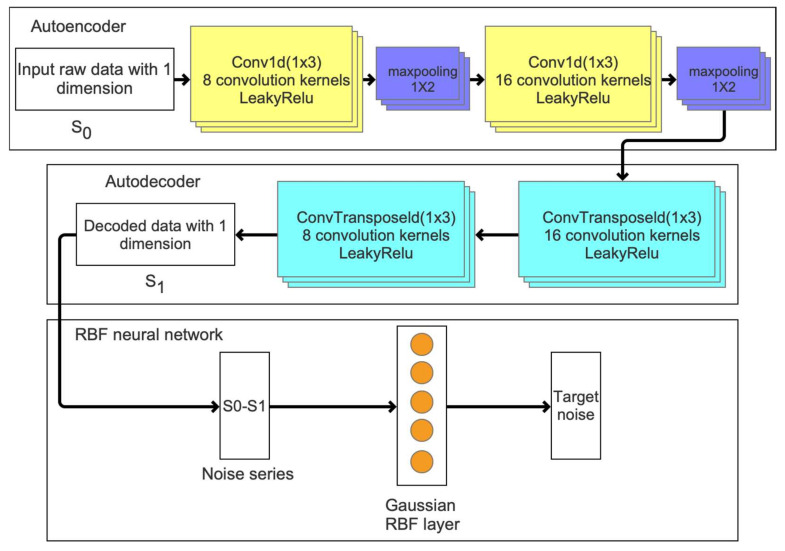
Framework of the noise optimization algorithm based on autoencoder and RBF neural network.

The advantage of denoising with the autoencoder is based on its feature extraction and data reconstruction capabilities, while the RBF network [34] can quickly model noise sequences using the less hidden layers and RBF function, i.e., Gaussian function. Considering that the goal of the EKF in this work is to optimize the **Q** and **R** matrices, the noise optimization algorithm based on the autoencoder and RBF (ARBF) neural network is proposed, as shown in Figure 4. The autoencoder [35] framework contains the autoencoder and autodecoder sections. The autoencoder section has a one-dimensional convolution of 1 × 3 for initial feature extraction from the input data sequence. To speed up data processing, eight convolution kernels are set in the first convolutional layer of the autoencoder section. The initial feature extraction sequences are processed by the max-pooling layer to reduce the network parameters and extract further feature information at the same time. Then, sixteen one-dimensional convolution kernels of 1 × 3 each are used to establish the feature information after the max-pooling layer, and the constructed autoencoder information is the output from the second max-pooling layer. The autodecoder section is designed symmetrically to reconstruct new data sequences. The LeakyRelu activation function [36] is applied to the autoencoder neural network, which initializes the convolutional network with an minimal initial value to avoid zero gradients during network operation.

The new data sequence generated by the autoencoder process is represented as S1, and the difference between S1 and initial input sequence S0 is the noise sequence. The noise sequence is converted as the input of the RBF neural network with the Gaussian kernel function as follows:

(7) $$K\left(x_{1},x_{2}\right)=\langle \phi \left(x_{1}\right),\phi \left(x_{2}\right)\rangle =\sqrt{\pi }\sigma \cdot exp\left[-\frac{{\left(x_{1}-x_{2}\right)}^{2}}{4\sigma^{2}}\right]$$

where $K\left(x_{1},x_{2}\right)$ represents the kernel function value of samples $x_{1},x_{2}$, which are expressed as Gaussian kernel functions of $\frac{1}{4\sigma^{2}}$. To extract the noise value in the unsupervised mode, the clustering method is introduced to train the input noise sequence. The clustering method is a commonly used unsupervised learning method [37]. In this work, the mutual reachable distance of each data point is calculated to obtain the target label as follows:

(8) $$d_{\mathrm{mreach}-k\;}\left(a,b\right)=\max\left\{core_{k}\left(a\right),core_{k}\left(b\right),d\left(a,b\right)\right\}$$

where $d_{\mathrm{mreach}-k\;}\left(a,b\right)$ is the mutual reachable distance of the samples *a* and *b*; $core_{k}\left(a\right)$ and $core_{k}\left(b\right)$ are the core distances of samples *a* and *b*, respectively; $d\left(a,b\right)$ is the Euclidean distance between samples *a* and *b*; and *k* is the smoothing factor. The mutual reachable distance enlarges the gap between the clusters, which provides a better clustering effect in theory. In this work, the cluster center value of the clustering method is the target noise, and the **Q** and **R** matrices of the EKF are constructed from the data noise sequence.

### 2.4. Experimental Method

**Figure 5:**
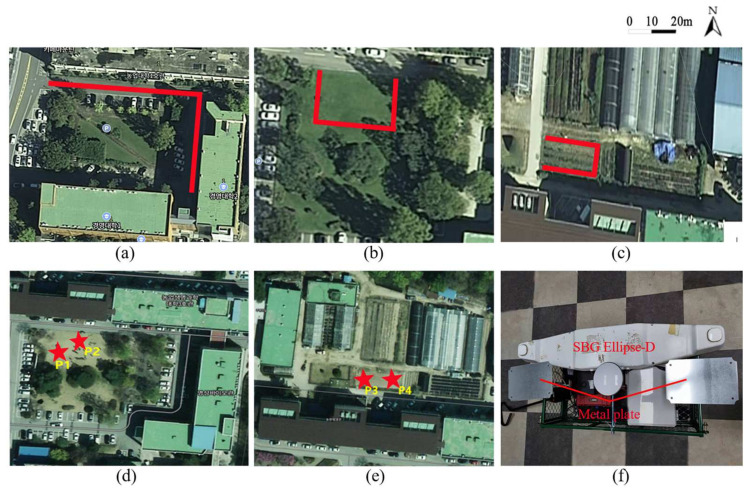
Experiment environment and path. (**a**) Experiment path on a road (length = 96 m). (**b**) Experiment path on grass (length = 60 m). (**c**) Experiment path in a field (length = 44 m). (**d**) Static experiments of P1 and P2. (**e**) Static experiments of P3 and P4. (**f**) SBG Ellipse-D and metal plates used in the experiments.

The experiments in this work were divided into three parts, i.e., initialization, static, and dynamic experiments. Ellipse-D (SBG Systems S.A.S., France), which provides high-accuracy RTK-GNSS data, was used as the baseline. The experiments were implemented at Kangwon National University, and the experimental scenes are shown in Figure 5. The red lines are the experimental paths. The initial test involved moving the AMR along the expected paths in Figure 5a–c. During the progress, data were collected and calculated according to the state and measurement equations. The **Q** and **R** matrix parameters were generated from the proposed multisensor fusion and ARBF algorithm with the acquired data sequences. The static and dynamic experiments were carried out based on the initialized parameters. The static experiments involved placing the AMR at points P1–P4 for 40 min, as shown in Figure 5d,e, to evaluate the static performance. The criteria for the static experiments are 50% circular error probability (CEP) and twice-the-distance root-mean-squared (2DRMS) values, whose formulas [38] are as follows:

(9) $$\mathrm{CEP}=0.589\left(\sigma_{x}+\sigma_{y}\right)$$

(10) $$2\mathrm{DRMS}=2\sqrt{\sigma_{x}^{2}+\sigma_{y}^{2}}$$

where $\sigma_{x}$ and $\sigma_{y}$ represent the Std of the UTM coordinates x and y.

**Figure 6:**
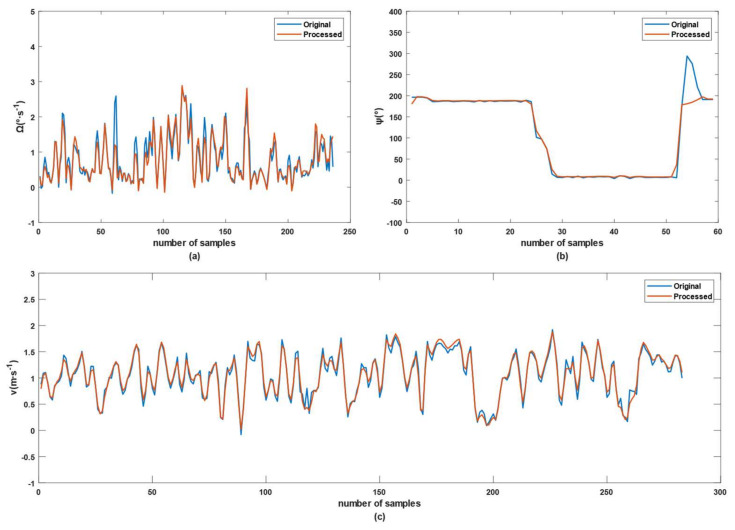
Graph of denoising results: (**a**) optimization results of Ω; (**b**) optimization results of ψ; (**c**) optimization results of *v*.

The dynamic experiments involved two sections, i.e., three different ground environments and a single abnormal sensor, to evaluate the position prediction performance under different conditions. The three different ground environments include road, grass, and field, as shown in Figure 6a–c, respectively. The single abnormal sensor condition contained data from RTK-GNSS with IMU and RTK-GNSS with encoder. In addition, to validate the performance of the proposed algorithm under the state that the RTK-GNSS data experienced interference, the metal plates were randomly applied to provide interference during the tests, as shown in Figure 5f. The RMSE was used in this work to evaluate the dynamic performance, whose formula is as follows:

(11) $$\mathrm{RMSE}=\sqrt{\frac{1}{N}\sum_{1}^{N}\left[{\left(x_{i}-x_{e}\right)}^{2}+{\left(y_{i}-y_{e}\right)}^{2}\right]}$$

where $x_{i}$ and $y_{i}$ represent the predicted position coordinates, and $x_{e}$ and $y_{e}$ represent the baseline positioning data from the Ellipse-D equipment.

## 3. Results

### 3.1. Noise Optimization Results Based on ARBF Algorithm

The noise optimization of the EKF based on the ARBF was mainly in terms of the initialization for multisensor fusion. After obtaining various data from the initialization process through the AMR, the autoencoder network generated a denoised data sequence. Then, the handled noise sequence from the denoised data was transferred to the RBF network to acquire the target noise value. Considering that the state and measurement equations contain many dimensions, Figure 6 shows only a graph of optimization results of Ω, ψ and *v* in the test area of Figure 5a. Among these three results, *v* is from the measurement equation, while Ω and ψ belong to the state equation. Thus, the parameters had a different number of samples.

The denoising result of Ω was shown in Figure 6a, and the convergence time was 6.6 s. The motion state of the AMR in the experiments contains drift noise, such as velocity and direction, resulting in spikes in the yaw-rate data. The autoencoder reduced the maxima of most of the spikes. The heading angle data in Figure 6b was also optimized in terms of the amplitudes of the instantaneous spikes, which may be generated by instantaneous changes in directions. The convergence time of Figure 6b was 4.1 s. The impact of noise on the system can be reduced, and the performance can be improved in the static and dynamic experiments. Figure 6c shows that the spikes in the original data were suppressed by the ARBF algorithm, of which convergence time was 7.3 s. The maxima of the optimized data were slightly lower than those of the original data, while the minimum of the valley was more significant than that of the original value. These results prove that the ARBF is effective at optimizing the noise of the AMR speed data. Although there is still little sample data increased, the main aim of this paper was to optimize the global data instead of local data. To quantify the noise optimization degree of the ARBF algorithm, the mean and Std of each dimension were analyzed, as given in Table 1.

As seen from Table 1, the ARBF algorithm plays a positive role in reducing the noise of each dimension. The ARBF had the smallest degree of noise optimization for position data because the RTK-GNSS data were calibrated with little noise. The denoising degree of $px_{k}$ was larger than the *x* coordinate of the measurement equation, while the denoising degree of $py_{k}$ was less than the *y* coordinate of the measurement equation. These results mean that additional noise was mixed in the AMR movement process, and the noise components were slightly different in the horizontal and vertical directions, which may be caused by physical properties, such as tire pressure and gravity center. The ARBF algorithm had the most significant effects on *v* of the state and measurement equations, whose Std values were reduced by 20.0% and 11.4%, respectively, and the mean values were reduced by only 0.23% and 0.33%, respectively. These results imply that the ARBF can suppress noise and slightly affect the mean value of the original data. Because displacement is essentially an integration of velocity over time, the mean value of the velocity in unit time directly affects displacement. The heading angle of the AMR is crucial to calculate the displacements in the X and Y directions. It is seen from Table 1 that the original heading angle difference between the state and measurement equations is 0.7961°, while the ARBF optimized angle difference is only 0.1°. Moreover, the Std of the two heading angles reduced by 1.83% and 0.9%. Thus, the noise in the heading angle was optimized with the ARBF algorithm. Hence, the noise sequence generated by the difference between the original and denoised data contains extracted features in each dimension. The target noise computed by the RBF network may thus have a positive impact on improving the position prediction performance of the multisensor fusion algorithm.

### 3.2. Results of Static Experiments

**Figure 7:**
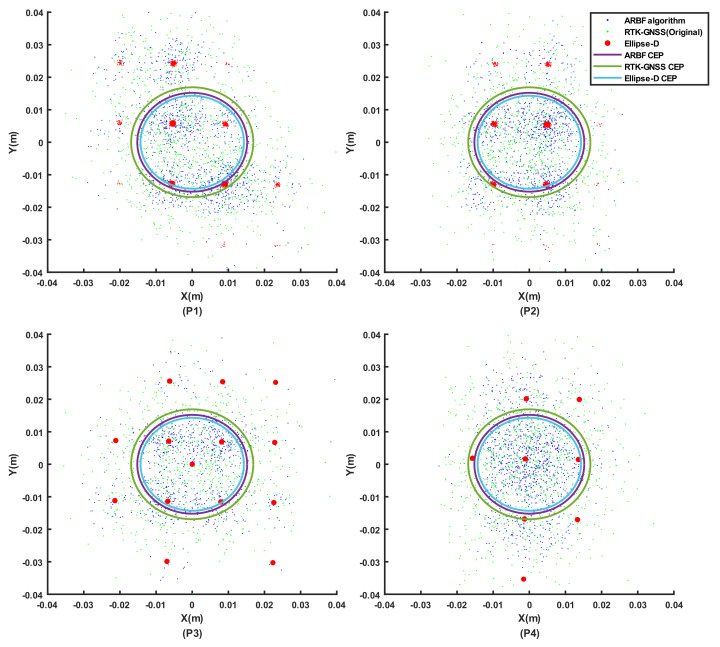
Results of static experiments: (**P1**) data points distribution for P1; (**P2**) data points distribution for P2; (**P3**) data points distribution for P3; (**P4**) data points distribution for P4.

Static experiments are essential for evaluating position prediction performance. The AMR was set at points P1–P4, whose results are shown in Figure 7. As seen from Figure 7, the positioning data of Ellipse-D and RTK-GNSS drift over time. The red points denote corrected position points from the Ellipse-D device so that the data were concentrated in special grids. The static performance of the ARBF is represented using the 50% CEP and 2DRMS metrics, as shown in Table 2. The CEP and 2DRMS of Ellipse-D were lowest with less noise and errors, thus reducing its positioning Std. The CEP and 2DRMS values of the ARBF algorithm decreased maximally by 25.9% and 24.4%, respectively. These results mean that the ARBF algorithm can optimize the noise and fluctuations of the system. The improved static performance has an important influence on the dynamic experiments.

### 3.3. Results of Dynamic Experiments

#### 3.3.1. Results of Position Predictions on Road, Grass, and Field

**Figure 8:**
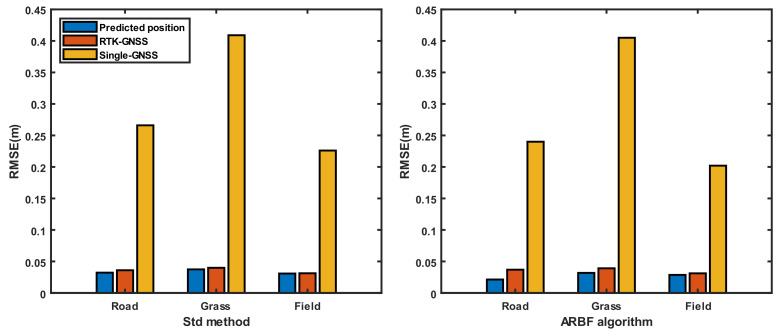
RMSE results of dynamic experiments in the three different environments.

**Figure 9:**
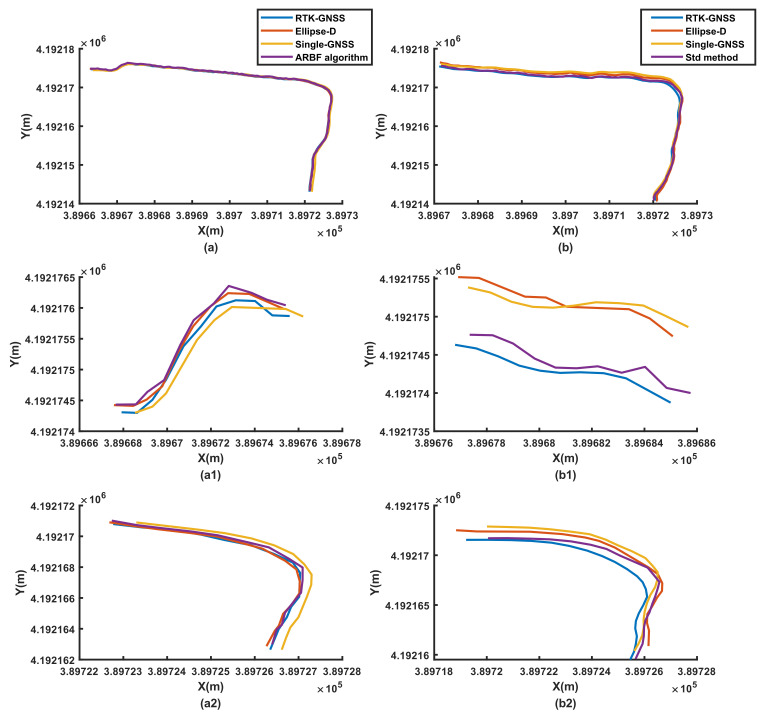
Experimental results from road: (**a**) position prediction results of ARBF algorithm; (**a1**,**a2**) local position data of (**a**); (**b**) position prediction results of the Std method; (**b1**,**b2**) local position data of (**b**).

**Figure 10:**
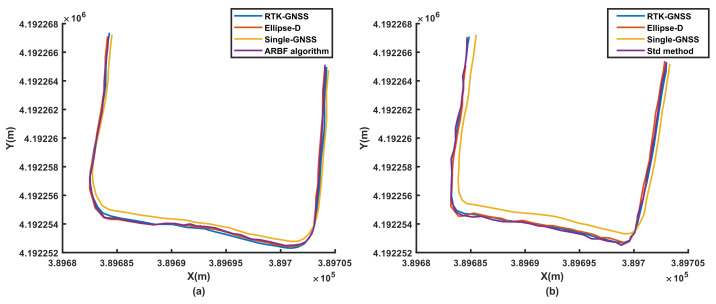
Experimental results from grass: (**a**) position prediction results of ARBF algorithm; (**b**) position prediction results of the Std method.

**Figure 11:**
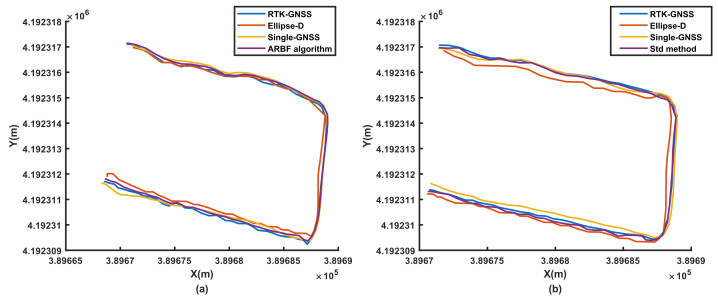
Experimental results from field: (**a**) position prediction results of ARBF algorithm; (**b**) position prediction results of the Std method.

To evaluate the performance of the ARBF algorithm under dynamic conditions, the system was first tested for the three different ground environments, i.e., road, grass, and field. At the same time, the noise matrix determined by the Std method was compared with that of the proposed ARBF optimization algorithm for the calculated root-mean-squared error (RMSE) of position prediction. The RMSE results are shown in Figure 8, and the dynamic motion paths are shown in Figure 9, Figure 10 and Figure 11, where the red paths represent the baseline data from the Ellipse-D equipment.

The RMSE range of the single-GNSS was 0.202–0.409 m for the three conditions, indicating that the single-GNSS had better position accuracy than the single-band GNSS with GNSS mode. The results in Figure 8 show that the ARBF algorithm provides high-accuracy position predictions for different ground environments, which are better than those of the common Std method. Because the real motion path of the AMR is not a straight line, the paths are also different. The results in Figure 9 are for the road environment, where Figure 9(a1,a2) are from Figure 9a. It is seen from the two subfigures that the ARBF can generate position predictions when the AMR turns with the heading angle. The RMSE in the local path is 0.0366 m, while the RMSE of the RTK-GNSS is 0.0676 m; that is, the accuracy of the proposed algorithm improves by 45.9%. Figure 9b represents the results of the Std method and its subfigures, i.e., Figure 9(b1,b2) are obtained from Figure 9b, which has the same local position as that in Figure 9a. The results show that the RMSEs of the Std method and RTK-GNSS are 0.0653 m and 0.0722 m, respectively; thus, the RMSE improved only by 9.56%. These results mean that the position prediction performance of the ARBF was significantly enhanced compared to that of the Std method. The results from grass and field environments are shown in Figure 10 and Figure 11, respectively. Both figures indicate that the ARBF algorithm works well, with RMSEs of 0.0368 m and 0.0201 m for grass and field, respectively. Owing to the uneven ground surface, the vibration noise increased, and the smoothness of the AMR carrier decreased, thereby causing more fluctuations in the experiment paths in the grass and field environments.

#### 3.3.2. Position Prediction of RTK-GNSS Signal with Interference

**Figure 12:**
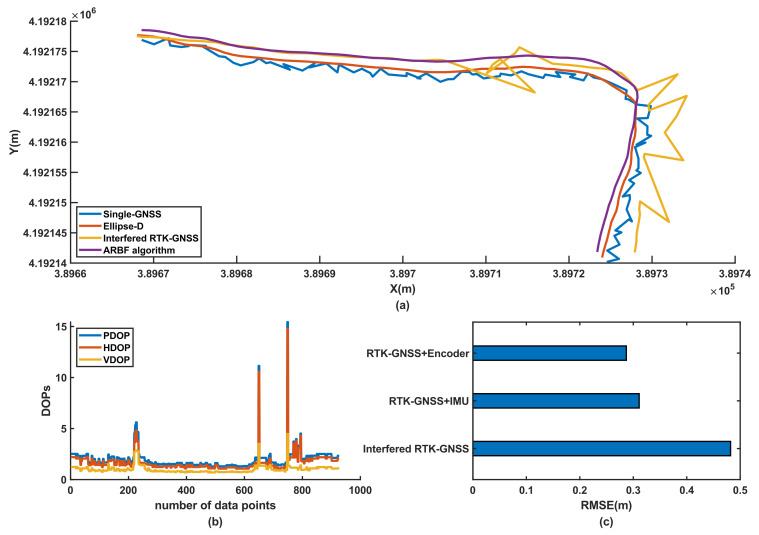
Experimental results of RTK-GNSS signal with interference: (**a**) position prediction results of the RTK-GNSS signal with interference; (**b**) DOP data of the RTK-GNSS in the experiment; (**c**) RMSE results of the experiment.

The signal of the RTK-GNSS was easily affected by the external environment, such as buildings and trees. To evaluate the position prediction robustness and performance of the system when the signal-to-noise ratio drastically changes in this work, RTK-GNSS signal with interference was simulated using the metal plates shown in Figure 5f, while the single-GNSS was not interfered with the metal plates; these results are shown in Figure 12a. In addition, to provide accuracy levels for the RTK-GNSS signal with interference, the dilutions of precision (DOP) data were collected with the $GNGSA data frame from the RTK-GNSS system during this experiment. The DOP data contain position dilution of precision (PDOP), horizontal dilution of precision (HDOP), and vertical dilution of precision (VDOP), which are shown in Figure 12b. The three-wave peaks in Figure 12b represent these three random interferences as instantaneous changes in the DOP. Although the positioning accuracy of the RTK-GNSS reduced to 1.216 m under these three interferences, as shown in Figure 12c, the position prediction accuracy of the ARBF algorithm still decreased to 0.245 m, which exceeded the RMSE of the single-GNSS case (0.482 m). The positioning accuracy of the RTK-GNSS may decrease significantly, which can lead to serious position deviations. With the ARBF algorithm, the position prediction performance of the AMR is improved. When the RTK-GNSS signal experiences interference within a short time, the system can still provide position estimations based on prior positioning as well as fused IMU and encoder sensor data to improve the overall position prediction accuracy.

#### 3.3.3. Position Prediction Results under Different Sensor Combinations

**Figure 13:**
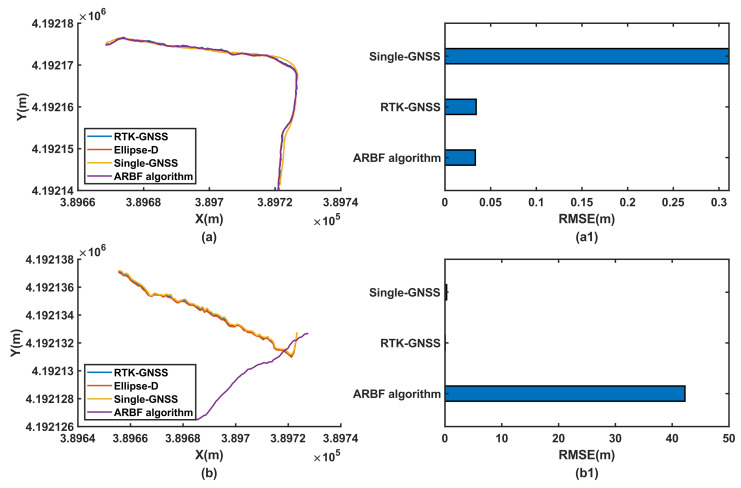
Position prediction results under sensor failure condition: (**a**) position prediction results of ARBF for RTK-GNSS and IMU case; (**a1**) RMSE results for RTK-GNSS and IMU case; (**b**) position prediction results of ARBF for RTK-GNSS and encoder case; (**b1**) RMSE results for RTK-GNSS and encoder case.

Sensor failure is a common phenomenon that seriously affects position prediction. These experiments were implemented on the road under encoder and IMU failures, and the results are shown in Figure 13 to evaluate the sensor fusion and system performance. Figure 13a shows that because the RTK-GNSS could provide basic position data and IMU could provide heading angle and acceleration data; the system could obtain integral acceleration data in the absence of encoder sensors, so that the ARBF algorithm could be implemented. However, in this experiment, the RMSE of the RTK-GNSS was 0.0341 m, while the RMSE of the ARBF was 0.0332 m, which is an improvement of only 2.6%. When all sensors work under normal conditions, the RMSE of the ARBF is improved by 23.7% compared to that of the RTK-GNSS. Figure 13b shows that the system cannot provide the predicted position without the IMU. Although rotary encoders can measure real-time velocities of the AMR, due to lack of heading angle and acceleration attitude data, the fusion algorithm loses odometry integration and heading angle measurement ability, resulting in position predictions that deviate from the actual direction of the AMR. The experiment results thus indicate that the IMU is crucial to position prediction in the ARBF and multisensor fusion system.

## 4. Discussion

In this study, the ARBF optimization algorithm shows good results for denoising. The denoised data sequences of the state and measurement equations of the EKF are analyzed using the mean and Std. The ARBF has little influence on the original data, i.e., the mean of each dimension does not change significantly, while the Std decreases, meaning that the dispersion degree of the original data also decreases. Denoising autoencoders are commonly used in data processing in many fields, such as image processing [39]. In this study, the denoised sequence was obtained from the autoencoder neural network constructed using a one-dimensional CNN. The main process included data feature extraction by the autoencoder CNN and reconstruction by the symmetric autodecoder network. Theoretically, the generated sequence is the original denoised data. Although the original data changes, the special features are preserved. At the same time, the unsupervised learning mode of the RBF neural network was applied to model the noise sequence to calculate the target noise as the noise matrix of the multisensor fusion algorithm. Thus, the ARBF indirectly achieves the purpose of dynamically determining the noise matrix, which positively influences position prediction. Goncalves et al. [28] developed a real-time classical stochastic model based on EKF and Gaussian distribution measurements error fusion to reduce the time to react so that the variance of position estimation was also reduced. Therefore, the ARBF algorithm in this paper could also be improved on the position estimation of robots in real-time. On the other hand, because the ARBF requires a significant amount of computing resources, it is important to deploy the model on a cloud server for remote interactions to improve real-time computing performance.

To evaluate the performance of the ARBF under the static state of the AMR, the 50% CEP and 2DRMS criteria were applied to determine the drift in position prediction. As classical accuracy indexes, these are widely used to determine navigation accuracies. Although the AMR was used under static conditions, the satellite signal was influenced by a variety of environmental factors, such as atmospheric conditions and buildings [40], thus resulting in position drift. Additionally, sensors such as the IMU may contain slight drift, which can cause drift in the position prediction of the multisensor fusion algorithm. The 50% CEP and 2DRMS values were reduced by 25.9% and 24.4%, respectively, with the ARBF algorithm compared to the RTK-GNSS. Static experiments were implemented at points P1–P4, where the results had slight differences because the sites had different horizontal planes. Therefore, further studies with the same environmental parameters may be carried out to acquire better results.

The dynamic experiments were thus carried out in the three different ground environments. The RMSE results of the ARBF and Std methods show reduced maxima by 23.7% and 9.56%, respectively, compared to the RTK-GNSS data. Although both methods were able to accurately predict positions, the ARBF showed better performance, especially in the AMR turning process. Several studies have reported multisensor fusion experiments based on the GPS/IMU/encoder. For example, Feng et al. [41] proposed a method to improve the robustness of the KF algorithm, and Liu et al. [42] developed an adaptive KF algorithm based on multiple sensors to optimize the position prediction ability of the navigation system. The improvements in the accuracies of the systems were limited under the low-accuracy GPS. With the increasing demand for high-accuracy navigation, the RTK-GNSS is expected to have positive effects on position prediction. On the other hand, it is important to evaluate the position prediction performance when the RTK-GNSS contains interference or when the sensors work abnormally. The results of Figure 12 indicate that the ARBF could still provide the predicted position data with poor RTK-GNSS signals. Although the RMSE increased, the prediction accuracy improved compared to that of the RTK-GNSS. The experiments involving lost encoder and IMU data show that the main effects of the encoder are to provide the velocity of the AMR for displacement integration to enhance the robustness of position prediction. When the IMU data are lost, the position prediction of the AMR shows serious deviations, indicating that the constructed system relies on the IMU sensor to obtain the motion attitude to achieve high-accuracy position prediction. This indirectly explains the importance of multisensor fusion for high-accuracy position prediction. However, the system presented herein for the AMR does not provide predictions of the heading angle, which is of great significance for navigation [43]. Thus, a possible improvement direction is to build a multisensor fusion optimization algorithm with a heading angle prediction function. Because the encoders are used to measure real-time velocities, the overall data output frequency can be increased through further study to satisfy the requirements of high-speed navigation and positioning. With the improvement in edge computing capabilities and increasing application of vision-based navigation, it is essential to combine the proposed system with vision navigation. In addition, because the AMR or vehicles may experience interference in long tunnels, with interruption of the RTK-GNSS or GNSS signals for different durations, it is important to study multisensor fusion algorithms [44] in such situations.

## 5. Conclusions

In this study, an improved AMR position prediction method based on the ARBF and multisensor fusion is proposed. The RTK-GNSS/IMU/dual encoders are used to construct a multisensor AMR, and a data collection system based on the Jetson Nano was developed simultaneously. Based on the proposed system, multisensor fusion based on the EKF and ARBF algorithm was established to improve the position prediction performance. The experiments indicated that the position prediction accuracies improved the maxima by 45.9% compared with the RTK-GNSS. At the same time, the ARBF algorithm could still provide position predictions when the RTK-GNSS signals experienced interference or when the dual encoder data were lost. Because position prediction can be affected by a variety of factors and the computational complexity of the ARBF algorithm, future research can consider optimizing the parameters of the fusion algorithm through remote interactions with the cloud server and study the position predictions when the RTK-GNSS experiences interferences of different durations.

## Figures and Tables

**Table 1:** Means and standard deviations of the original and optimized data for each dimension.

Vectors	Dimensions	Mean		Std	
		Original	Denoised	Original	Denoised
$X_{k}$	$px_{k}$ (m)	389,685.3841	389,685.3164	27.3088	27.2844
	$py_{k}$ (m)	4,192,301.2622	4,192,301.2380	29.1503	28.8584
	$\psi_{k}$ (°)	112.9747	111.9958	92.7408	91.0441
	$v_{k}$ (m·s^−1^)	2.9260	2.9193	0.4964	0.3973
	$\Omega_{k}$ (°·s^−1^)	0.8234	0.8188	0.8609	0.7872
	$a_{k}$ (m·s^−2^)	0.0090	0.0071	1.0978	0.9914
$z_{k}$	x (m)	389,685.2175	389,685.1613	27.3992	27.3089
	y (m)	4,192,301.1060	4,192,300.8611	17.0727	16.7711
	ψ (°)	112.1786	111.8958	92.7854	91.9487
	v (m·s^−1^)	2.9062	2.8965	0.4883	0.4326
	a (m·s^−2^)	0.0087	0.0086	0.8847	0.8602

**Table 2:** Results of 50% CEP and 2DRMS.

Experiment Site	Criteria	RTK-GNSS (m)	ARBF (m)	Ellipse-D (m)
P1	50% CEP	0.0169	0.0152	0.0143
	2DRMS	0.0410	0.0370	0.0349
P2	50% CEP	0.0152	0.0126	0.0115
	2DRMS	0.0370	0.0310	0.0285
P3	50% CEP	0.0156	0.0127	0.0126
	2DRMS	0.0376	0.0307	0.0306
P4	50% CEP	0.0158	0.0117	0.0105
	2DRMS	0.0385	0.0291	0.0264

## Data Availability

The data can be requested from the corresponding authors.
